# Nitrate of Silver

**Published:** 1899-02

**Authors:** B. F. Arrington


ARTICLE VI.

### NITRATE OF SILVER.

DR. B. F. ARRINGTON.

Not only possibly, but in all probability nitrate of silver is the best and most reliable of all known microbe and germ destroying remedies, and is the best and surest of all remedies for checking caries and for preserving teeth superficially affected.

It is questionable if any remedy is so essential and so effective for the preservation of the temporary teeth as nitrate of silver. If children's teeth are watched carefully and frequently, and treated properly with nitrate of silver, they will not need to be prematurely extracted, nor will they need filling.

Caries timely treated with this remedy (powerful in effect, but harmless) can be checked and teeth preserved to better advantage under some circumstances than by filling, and with economy of time, less cost and infinitely less discomfort to patients.

The occasional unsightly discoloration produced by application of the remedy should not be seriously objected to, when the comfortable preservation of the teeth is under consideration. The object in view and possible results attainable are first to be considered.

Our every action in practice should conform strictly to principles of true conservatism (human organs and the human system require it) whether profits or losses follow. The preservation of the natural teeth through the most thorough line of conservative treatment should, in my judgment, be our governing principle. With such a base for action, results following will be satisfactory, and true science in management and safe-guarding oral surgery will be advanced and strengthened.

There are several reasons why it is right and proper to preserve the temporary teeth, if possible, without excavating and filling.

Liberal experimenting for results should be practiced, not only in treatment of the temporary teeth, but with the permanent also. By judicious application of nitrate of silver the ravages of decay can be effectually checked, and teeth preserved for years in a comparatively normal state of appearance and comfort. No one can fully realize the results to be derived in the use of any remedy without freely experimenting.

If a cavity has jagged edges, trim and smooth with burs, chisels or rightly shaped excavators, as judgment directs, also the walls and base of cavity, and make as self-cleaning and as smooth as possible, that the remedy may be effectively placed in contact with the entire surface of cavity. Let the material be finely crushed, twirl cotton on an old discarded hand bur of suitable size, moisten with a saturated solution of the material, then touch to the crushed nitrate of silver and carry to the cavity or surface, twirling and holding in place for some moments, always having cavities dry, and using the precaution to protect the gums with bituminous paper before applying the remedy, and as a safeguard against injury to the mucous membrane by excess of quantity; keep at hand a saturated solution of table salt, which is an excellent antidote and easily applied by taking into the mouth and holding a few seconds. As soon as the nitrate of silver is applied to a cavity, pack tightly with cotton for several minutes, then remove and wash cavity thoroughly. The day following, or within a few days, smooth and polish cavity as may be necessary, and re apply the remedy as previously, and dismiss. The work is finished, and the chances are nineteen to one against recurrence of decay, unless the tooth is of very soft structure, then repeat the treatment, and all will go well, and no filling in question to produce unpleasant effects through thermal charges.

Saving of time, expense and discomfort to patients, in treating for preservation of teeth should be rightly considered, and practice regulated accordingly. The interest and welfare of patients is first to be considered, then the dentist.

I hold to the idea that the ultimate zenith of the seience of dentistry will consist in the successful treatment and preservation of teeth without necessity for excavating and filling, or the operation of extracting, except in accident or overcrowding of teeth. Such is possible if we will halt for reflection, then start on a right line of practice, and let our teaching and practice be strictly in accord with nature's laws, as in the treatment of other organs of the body. There is no reason why it should be otherwise.

Had the idea advanced and advocated by Dr. Chapin Harris, prior to the establishment of the Baltimore Dental College, to create a Chair of Oral Surgery in every medical college, it is possible there would not now be one tooth extracted where there are a thousand, and plates, bridges or crowning would be but seldom called for. Comfort would be realized through the blessing of health; and well preserved teeth would be much greater than at present.

We are reaching out too rapidly and too far. It will be best to draw in a little, until within legitimate limits, and regulate our practice of oral surgery for health of mouth and preservation of teeth, on a true line of conservatism. Possibly those we serve will be better served, and oral surgery as a specialty will be more favorably esteemed.—*Dental Brief.*

